# Group B Streptococcus Osteomyelitis in a Healthy Adolescent

**DOI:** 10.7759/cureus.10798

**Published:** 2020-10-05

**Authors:** Andrew Wahba, Rafik ElBeblawy

**Affiliations:** 1 Pediatrics, University of Texas Health Science Center at Houston McGovern Medical School, Houston, USA; 2 Infectious Diseases, University of Louisville School of Medicine, Louisville, USA

**Keywords:** group b streptococcus, osteomyelitis, adolescent

## Abstract

Group B Streptococcus (GBS), Streptococcus agalactiae, is a bacterium often screened for pregnant women and associated with neonatal infections. However, GBS disease is also rising among non-pregnant adults, especially among immunocompromised patients. The median age of non-pregnant adults with invasive GBS disease is 64 years. It can present as skin and soft tissue infection, osteomyelitis, pneumonia, urosepsis, and meningitis. There is very limited data on GBS disease occurring in the pediatric population past the infancy stage. In this report, we present a case of a 16-year-old male with GBS osteomyelitis.

## Introduction

Group B Streptococcus (GBS or Streptococcus agalactiae) is a gram-positive cocci that frequently colonizes the human genital, gastrointestinal and upper respiratory tracts. Though decreased incidence due to wide administration of intrapartum antibiotics [1], they are associated with neonatal infections either in utero or during delivery in the form of sepsis, pneumonia, or meningitis [2]. A markedly increasing population as a new target for GBS are non-pregnant adults. Epidemiological surveillance identified invasive GBS incidence among non-pregnant adults increased significantly by 34% from 8.1 cases per 100 000 population in 2008 to 10.9 in 2016; incidence was highest among age 65 years or older [3].

Patient populations with underlying medical conditions are reported to be the most vulnerable for GBS infection, especially poor glycemic control and obese patients. Bacteremia without focus, skin and soft tissue infections are the most predominant sites of clinical infections in non-pregnant adults [3,4]. While osteomyelitis represents 15% of cases in late childhood to elderly patients [3].

Literature has very scarce reports for defining the invasive GBS diseases in infants older than 90 days and during childhood. In this case report, we present a 16-year-old otherwise healthy male with GBS osteomyelitis.

## Case presentation

A 16-year-old African American male with a history of fibroma of the right knee presented to our emergency department with a three-week history of right knee pain, preceded by five days of subjective fever. Pain was severe and limited his ability to walk. Otherwise, the patient denied any history of trauma. He has no significant past medical or surgical history except for fibroma of the right knee and leg length discrepancy (unclear etiology if it was congenital or caused by fibroma). He had a basketball injury four years ago and a knee X-ray at that time showed non ossifying fibroma. No family history of bone diseases or sickle cell disease. The patient lives at home with his parents and usually plays basketball. He denied smoking, alcohol, or drug use, and no history of sexual activity was identified.

On physical examination, oral temperature was 98°F, heart rate 99 beats per minute, blood pressure 129/60 mmHg, respiratory rate 16 times per minute, and oxygen saturation was 100% on room air. Weight was 56.4 kg with body mass index (BMI) 21. The right knee exam showed mild edema, but no tenderness, warmth nor erythema. He was able to flex his legs about 30 degrees when supine. No signs of marked stiffness or joint deformities were evident in other joint examinations. Heart and lung exams were clear, and no skin rash or lesions were seen. The patient looked otherwise well developed with appropriate mood and affect.

Initial clinical laboratory assessment revealed elevated erythrocyte sedimentation rate (ESR) >100 mm/hr and c-reactive protein (CRP) 91.2 mg/L. Normal white blood cell (WBCs): 8.1 x10^3^/µL. Mild decreased hemoglobin and hematocrit values, 10.4 g/dl, and 30.9%, respectively (Table 1). 

**Table 1:** Initial laboratory values WBC, white blood cell; RBC, red blood cell; MCV, mean corpuscular volume; RDW, red blood cell distribution width; INR, international normalized ratio; APTT, activated partial thromboplastin time; BUN, blood urea nitrogen; ALT, alanine aminotransferase; AST, aspartate aminotransferase; Alk Phos, alkaline phosphatase; LDH, lactate dehydrogenase; CRP, c-reactive protein; ESR, erythrocyte sedimentation rate.

	Intial presentation
WBC x 10^3^/mm^3^	8.1
RBC x 10^6^/mm^3^	3.74
Hemoglobin g/dL	10.4
Hematocrit %	30.9
MCV fL	82.6
RDW %	13.3
Platelet x 10^3^/mm^3^	412
Neutrophil %	67.2%
Lymphocyte %	24.7%
Esinophil %	2.3%
Neutrophil x 10^3^/mm^3^	5.4
Monocytes x 10^3^/mm^3^	0.4
Lymphocyte x 10^3^/mm^3^	2
Protime seconds	15.2
INR	1.2
APTT seconds	29.8
Sodium mEq/L	134
Potassium mEq/L	3.3
Chloride mEq/L	99
CO2 level mEq/L	26
Anion gap mEq/L	12.3
Creatinine mg/dL	0.72
BUN mg/dL	14
Calcium mg/dL	9.8
Phosphorus mg/dL	4.3
ALT unit/L	14
AST unit/L	16
Alk Phos unit/L	105
LDH unit/L	131
CRP mg/L	91
ESR mm/hr	>100

**Figure 1:**
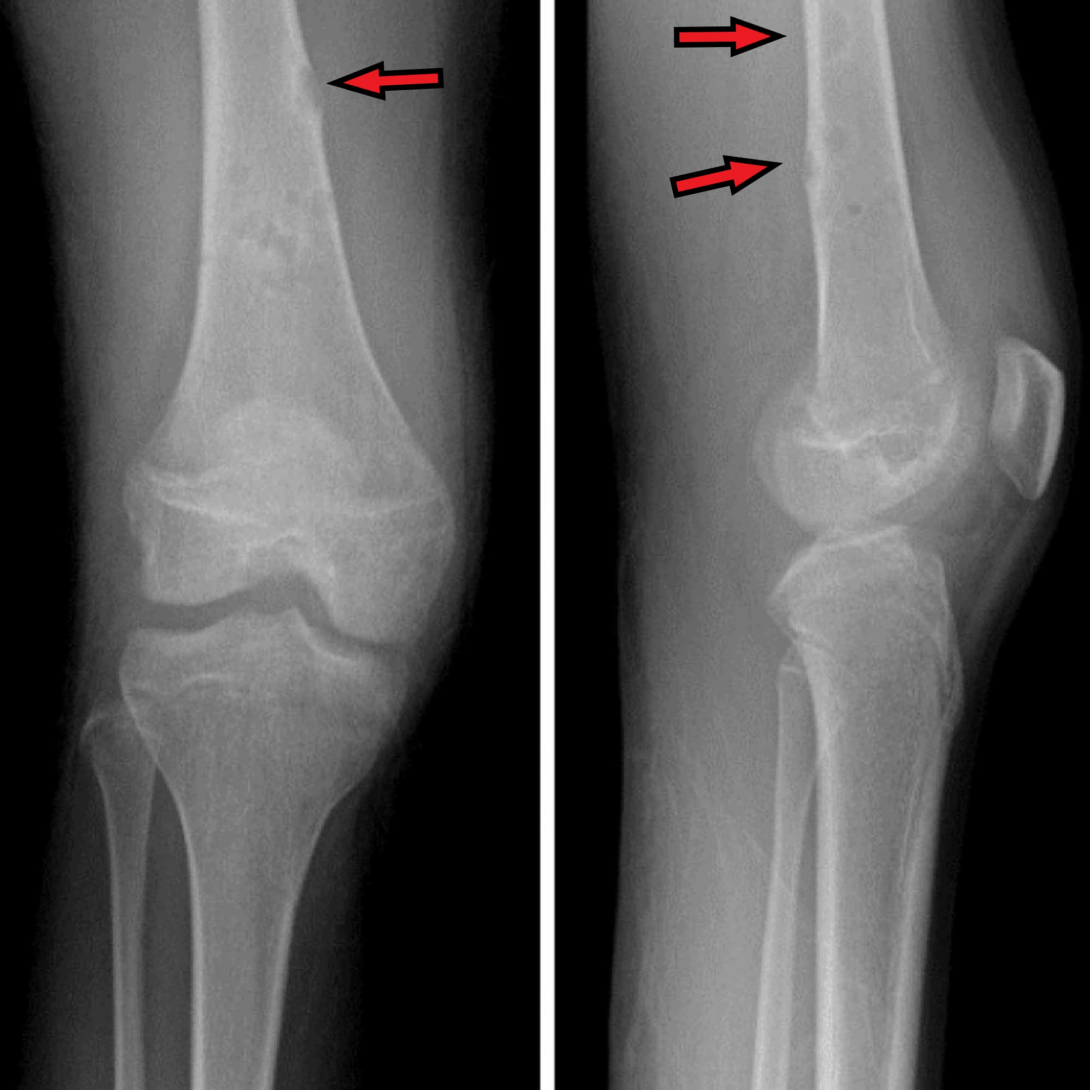
X-ray of the right knee two views (anteroposterior and lateral view) Red arrows showing lytic lesions of the distal femoral metadiaphysis with surrounding reactive sclerosis

X-ray of the right knee showed lytic lesions; there were multiple cortically based ill-defined lytic lesions of the distal femoral metadiaphysis with surrounding reactive sclerosis (Figure 1). The most proximal lesion was eccentrically located and demonstrated cortical thinning with questionable cortical breakthrough.

**Figure 2:**
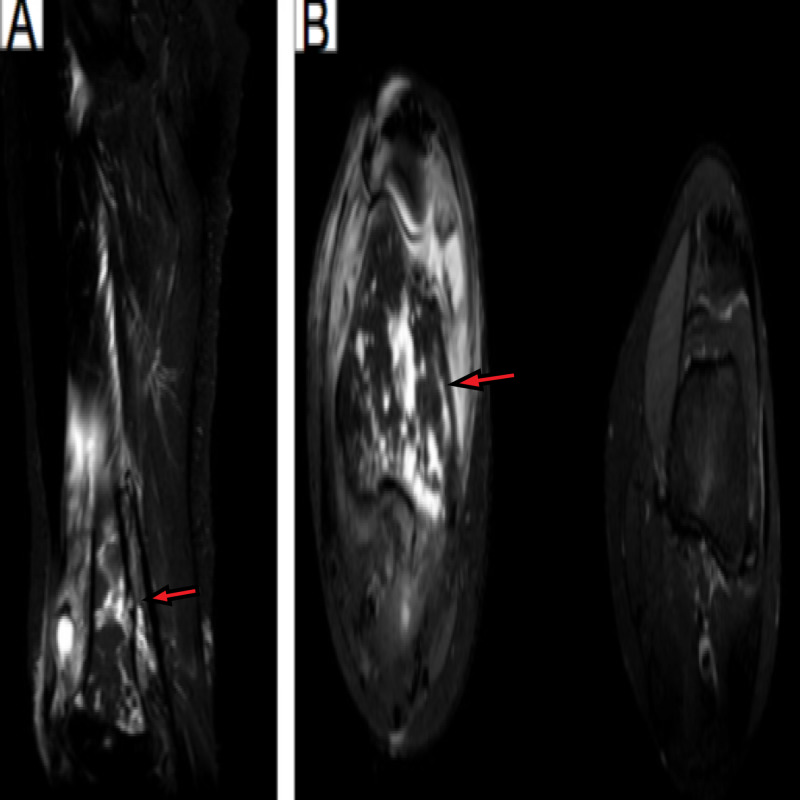
(A) coronal MRI image of the right knee (B) axial MRI image of the right knee compared to the left knee Red arrows showing scattered enhancing areas with cystic changes involving the distal femur with extensive myositis.

MRI of the right knee showed scattered enhancing areas of marrow edema and cystic changes involving the right distal femoral diaphysis, metaphysis, and epiphysis (Figure 2). Also, there was extensive cellulitis and myositis surrounding the right knee and extended proximally to the level of the greater trochanter with small developing organized fluid collections within the proximal vastus lateralis and intermedius muscles, representing evolving abscesses.

Because of the lytic lesions noted on imaging, bone biopsy, and knee aspiration were subsequently performed. Synovial fluid analysis revealed hematogenous cloudy fluid, elevated WBCs 4,000/µL, and >90% of polymorphonuclear leukocytes (PMN) (Table 2).

**Table 2:** Synovial fluid analysis

Test	Value
Glucose mg/dL	54
Protein g/dL	7.6
White blood cells (WBC) /mm^3^	4000
polymorphonuclear leukocytes (PMN)	92%
Lymphocyte	3%
Macrophage	5%
Clarity	cloud

On day three, he underwent debridement and irrigation of the right knee in the operating room. Operative findings revealed large amounts of thick sanguineous fluid, as well as necrotic fat, and thick gelatinous fluid was obtained with purulent material. The patient was then started on intravenous (IV) ceftriaxone awaiting culture results. Joint fluid culture grew GBS sensitive to ceftriaxone. Bone biopsy showed acute on chronic osteomyelitis with infiltration of histiocytes and plasma cells. Synovial biopsy revealed fibro-purulent debris and focal granulation tissue. 

On day seven, he was discharged to complete a four-week course of IV ceftriaxone followed by four weeks of amoxicillin. CRP and ESR values showed down-trending which normalized by day 70 (Table 3).

**Table 3:** Trending inflammatory markers from day 1 (initial presentation) to day 70 (antibiotic course completion) CRP, c-reactive protein; ESR, erythrocyte sedimentation rate

	CRP mg/L	ESR mm/hr
Day 1	91	>100
Day 3	109	>100
Day 4	126	>100
Day 7	82	>100
Day 8	52	>100
Day 40	4.3	17
Day 70	3	5

Given the presence of histiocytes on bone biopsy, he was referred to oncology for further workup of Langerhans cell histiocytosis.

## Discussion

GBS or Streptococcus agalactiae is a gram-positive cocci most commonly known as a colonizer of the genital and lower gastrointestinal tracts in humans. It had the highest prevalence of invasive diseases in neonates and pregnant women before the wide antenatal screening and administration of intrapartum antibiotic prophylaxis in indicated obstetric conditions.

Identification of non-pregnant adults with GBS invasive disease has gained more interest in the past 20 years with evident increasing incidence in elderly patients with at least one associated comorbidity in 95% of cases [3-5]. In 2016, 3146 cases were identified in non-pregnant adults, 48% ≥ 65 years, and only 18% were black. Age group 18-39 represented 7.6% of cases, 35.6% were black. The overall case fatality rate declined from 7.5% to 5.6% in 2016 and reported the lowest in the age group of 18-39 years by 3% compared to older patients [3]. 

In a surveillance study for the spectrum of invasive GBS disease in the US from 1999-2005, Phares et al. identified 233 childhood cases (age 90 days-14 years) out of 14,573 cases with invasive GBS disease. 60% of the childhood cases occurred before 12 months of age and only 90 non-infant cases (age 1-14) were identified throughout the surveillance (0.6%). However, in adults (age 15 years or older), more than 90% of cases were reported in patients 65 years or older. The incidence ratio for patients 15-64 years old was 5.0 compared to 26.0 per 100,000 cases in 65 years or older patients [5].

The most common associated comorbidities in infected patients are diabetes mellitus and obesity. Patients with extremes of body mass index are more susceptible to infection and those with HbA1c ≥9.5% show a four-fold greater risk for GBS invasive disease [3,4,6]. Other associated underlying conditions in more than 15% of patients were cancer, chronic skin diseases, heart failure, atherosclerotic cardiovascular, chronic renal and neurological diseases. In childhood invasive GBS disease, described by Phares et al., 44% of infected patients aged one to 14 years had at least one underlying medical condition [5]. 

Bacteremia without focus, skin, and soft tissue infections are the most predominant sites of infections in non-pregnant adults [3,4]. Osteomyelitis and septic arthritis were identified in 15.1% and 5.4% of patients (age 18-39) respectively [3]. Childhood invasive disease (age 90 days-14 years) manifested with bacteremia without focus in 58% of cases, while septic arthritis represented only 5% [5]. GBS osteomyelitis most often occurs by contiguous spread or direct inoculation. GBS septic arthritis is usually monoarticular with the most affected sites are the knee followed by the shoulder and ankle joints [7]. Patients with GBS septic arthritis tend to have significantly higher CRP, longer hospital stay, and length of treatment but lower mortality rate and limb loss compared to non-GBS septic arthritis [8]. 

GBS strains are classified into different serotypes based on variances in capsular polysaccharides structure, with serotypes Ia, Ib, II, III, and V being most prevalent [9]. GBS isolates are most susceptible to penicillin G, ampicillin, and cephalosporins. 

This article was persented as an abstract (Abstract: Meraj A, Wahba A, Balachandran A, Hsu J. Group B Streptococcus: A Culprit in More Than Just Infants. 2020 Southern Regional Meeting; August 2020) https://jim.bmj.com/content/68/2/435.1?

## Conclusions

Invasive GBS infection is rare in the adolescent population. Pediatricians should rule out underlying medical conditions that may have increased their patient’s susceptibility to developing the disease.

## References

[1] Schrag SJ, Verani JR (2013). Intrapartum antibiotic prophylaxis for the prevention of perinatal group B streptococcal disease: experience in the United States and implications for a potential group B streptococcal vaccine. Vaccine.

[2] Madrid L, Seale AC, Kohli-Lynch M (2017). Infant group B Streptococcal disease incidence and serotypes worldwide: systematic review and meta-analyses. Clin Infect Dis.

[3] Francois Watkins LK, McGee L, Schrag SJ (2019). Epidemiology of invasive group B Streptococcal infections among nonpregnant adults in the United States, 2008-2016. JAMA Intern Med.

[4] Skoff TH, Farley MM, Petit S (2009). Increasing burden of invasive group B Streptococcal disease in nonpregnant adults, 1990-2007. Clin Infect Dis.

[5] Phares CR, Lynfield R, Farley MM (2008). Epidemiology of invasive group B Streptococcal disease in the United States, 1999-2005. JAMA.

[6] Jump RLP, Wilson BM, Baechle D (2019). Risk factors and mortality rates associated with invasive group B Streptococcus infections among patients in the US veterans health administration. JAMA Netw Open.

[7] Nolla JM, Gómez-Vaquero C, Corbella X (2003). Group B streptococcus (Streptococcus agalactiae) pyogenic arthritis in nonpregnant adults. Medicine (Baltimore.

[8] Wang VTJ, Tan JH, Pay LH, Wu T, Shen L, O'Neill GK, Kumar VP (2018). A comparison of Streptococcus agalactiae septic arthritis and non-Streptococcus agalactiae septic arthritis. Singapore Med J.

[9] Raabe VN, Shane AL (2019). Group B Streptococcus (Streptococcus agalactiae). Microbiol Spectr.

